# Department of Therapeutics

**Published:** 1893-03

**Authors:** David Cerna

**Affiliations:** Demonstrator of Physiology in the Medical Department of the University of Texas, etc.


### DEPARTMENT OF THERAPEUTICS.

CONDUCTED BY DAVID CERNA, M. D., PH. D.,
Demonstrator of Physiology in the Medical Department of the University of Texas, etc.

ANAGYRINE.—At a recent meeting of the Societé de Biologie, Gley (*La Semaine Medicale*, 1892) reported his studies on this alkaloid, obtained from the *Anagyris fœtida*. He finds that the drug slows the heart, but at the same time increases the arterial pressure, even after section of the spinal cord. The action of the alkaloid is, however, prevented by chloral. The author, finally, concludes that, judging from the antagonism between these two drugs, *anagyrine* acts principally on the peripheral ganglia.

THE PREVENTION OF ASIATIC CHOLERA.—In an excellent short article on the cause and prevention of this disease, Frederick Gaertner (*International Medical Magazine*, January, 1893), says: "There is no doubt that the only means of preventing the spread of a contagious or infectious disease, is by the complete isolation of the victim and the prevention of any contact whatsoever with any article that may contain the germ. By this I mean effective quarantine. * * * All precautionary sanitary and protective measures should be scrupulously applied, and all examinations of excreta and secreta be continued until not a single comma bacillus has been discovered for eight or ten days. Then and then only can we be assured that cholera has been excluded and an epidemic avoided. Let all strictest sanitary measures be employed, and quarantine established at every seaport, State line, or even at the city limits; let all the traveling public be closely examined, and all effects subjected to a careful process of cleansing, fumigatlon and disinfection. Let the dead be cremated, and all the articles that have in any way come in contact with the disease be burned. All luggage, including the mails, should be exposed to an intense degree of heat, say 220° F., for two or three hours' duration, and then to extreme cold, say zero F., for a period of time from six to twelve hours. If these means are employed it will be impossible for any germ or micro-organism to exist, or be in a state of active development of the disease. Thus and thus only can we protect our people from the awful slaughter which this dread disease would certainly effect, were it to gain an entrance into America, and which we might anticipate in the very near future unless strict sanitary and preventive measures are adopted."

---

COCAINE IN TINNITUS AURIUM.—Carralero (*El Siglo Médico*, No. 14, 1792, Madrid) calls attention to the good effects of cocaine in tinnitus aurium, due to alterations of the tympanum, the buzzing sound of which in the ear is extremely painful to the patient. The author instills a solution of cocaine of the strength of four per cent., into the tympanum through the Eustachian tube, especially when the buzzing follows hyperæmia and catarrh of the tympanum, congestion of the labyrinth, a hyperæsthetic condition of the internal or middle ear, "sclerotic" otitis, and other pathological conditions. The results of this medication have been most excellent. According to the author these instillations should be practised at the beginning every day, and afterwards every two or three days. Carralero employed the sound invented by Itard.

---

THE ANTIDOTES TO PHOSPHORUS.—In an able experimental research, E Q. Thornton (*Therapeutic Gazette*, January 16, 1893) arrives at interesting conclusions, from a practical point of view. His experiments were performed on dogs. He found that in all cases of phosphorus-poisoning, in which the *sulphate of copper* was used as an antidote, death resulted! Although the animal to which the copper salt alone was given recovered, decided gastro-enteritis resulted, and, therefore, the author concludes (with much reason) that this drug should cease to be recommended in phosphorus-poisoning. He tried *peroxide of hydrogen*, but found this agent too slow in oxidizing the phosphorus, and too irritating upon the digestive tract to be a valuable antidote. The author also believes that inasmuch as the old French oil of turpentine cannot be obtained, this agent should likewise cease to be considered as a practical antidote.

We may say in this connection that Antal (*Lancet*, June 4, 1892), by experiments on animals, has found the *permanganate of potassium* antidotal in acute phosphorus-poisoning. Hajinos (*Ibid*) has employed it with success in some cases which have come under his care in the Rochus Hospital at Buda-Pesth. One case was that of a patient who drank a solution of phosphorus made from two matches, and was immediately taken into the hospital. The stomach was washed out, and within half an hour of swallowing the poison, Hajinos introduced 500 grammes of a one-tenth per cent. solution of the potassium permanganate into the stomach of the patient. There was no vomiting or pain, and the next day, as the man felt quite well, he left the hospital.

---

THE THERAPEUTIC USES OF PIPERAZIN.—David D. Stewart (*Therapeutic Gazette*, January 16, 1893) contributes a most valuable paper on the above subject, detailing at length three cases in which the drug was used. The patients were suffering from stone in the bladder. In two of the cases a cure was practically established. In one of these two cases all the symptoms referable to the kidney had been absent for several months, notwithstanding that piperazin had been discontinued for six months; and another important fact was, that the permanent disappearannce of gravel from the urine, a symptom which, previous to the administration of the drug, had been persistent and troublesome. Similar results were obtained in case 2, in which the former attacks of lumbar pain, of seven years' continuance, had been absent for seven months under the influence of piperazin. In the third case, one of probable mulberry calculus, no benefit was obtained from the employment of the drug. In these cases, neither the acidity of the urine nor an increase in the excretion of urea was appreciably affected. In only one of these instances was the amount of urine increased, although the author states that in most cases the drug augments the secretion of this fluid.

---

SALOPHEN AS AN ANTIRHEUMATIC.—In a preliminary note, H. A. Hare (*Therapeutic Gazette*, January 16, 1893) reports four cases of rheumatism in which salophen gave satisfactory results. The author asserts that in these cases, as well as in others in which he has employed the drug, it has given results which are so good that he has added the remedy to the list of other agents which he constantly bears in mind as meeting important indications. He has also found salophen of value in a host of other ailments closely allied to true rheumatism, and in which it has given him the best results.

---

THE VALUE OF COCAINE IN CATARRHAL AFFECTIONS OF THE UPPER AIR PASSAGES.—Clarence C. Rice (*New York Medical Journal*, January 21, 1893) in an interesting article on the value of sprays in the treatment of catarrhal affections of the air passages, states that cocaine is of great service; that a very weak percentage of it, say one-half of one per cent., added to astringent sprays, will to a great extent nullify the first irritating effect of the topical application. Mild solutions of cocaine, that is, less than one per cent., are perhaps as useful astringents as can be employed, and none of the uncomfortable reactions which sometimes follow the stronger solutions, are seen. These mild cocaine solutions seem to clinch the beneficial effects of astringents when used in combination. Cocaine seems to have justly supplanted solutions of opium, morphine, and bromide of potassium, and the author knows of no beneficial effect to be obtained from aconite preparations which cocaine does not more surely afford. The strength of the cocaine solutions can be more easily regulated than those of opium and aconite. The writer believes that cocaine sprays have rendered laryngeal operations and manipulation easy, and have saved life in relieving laryngeal dyspnœa, until obstructions could be removed.

